# Bone Density, Geometry, and Mass by Peripheral Quantitative Computed Tomography and Bone Turnover Markers in Children with Diabetes Mellitus Type 1

**DOI:** 10.1155/2022/9261512

**Published:** 2022-04-18

**Authors:** Maciej Jaworski, Elżbieta Wierzbicka, Edyta Czekuć-Kryśkiewicz, Paweł Płudowski, Maria Kobylińska, Mieczysaw Szalecki

**Affiliations:** ^1^Department of Biochemistry, Radioimmunology and Experimental Medicine, The Children's Memorial Health Institute, Warsaw, Poland; ^2^Department of Human Nutrition, Warsaw University of Life Sciences-SGGW, Warsaw, Poland; ^3^Department of Endocrinology and Diabetology, The Children's Memorial Health Institute, Warsaw, Poland; ^4^Faculty of Medicine and Health Sciences, Jan Kochanowski University, Kielce, Poland

## Abstract

**Background:**

The type 1 diabetes mellitus (T1DM) is a chronic systemic autoimmune-mediated disease characterised by the insulin deficiency and hyperglycaemia. Its deleterious effect on bones concerns not only bone mass, density, and fracture risk but also may involve the linear growth of long bones. Studies on the lower leg in children with T1DM by pQCT have generated conflicting results, and most of the studies published so far focused only on a selected features of the bone. An additional information about growth, modelling, and remodelling processes can be gathered by the bone turnover marker measurement. The objective of the study was to evaluate bone mineral density, mass, and geometry using peripheral quantitative computed tomography as well as bone turnover markers in the patients with type 1 diabetes mellitus. *Material and Methods*. Bone mineral density, mass, and geometry on the lower leg using peripheral quantitative computed tomography and serum osteocalcin (OC) and carboxyterminal cross-linked telopeptide of type 1 collagen (CTx) were measured in 35 adolescents with T1DM (15 girls) aged 12.3-17.9 yrs. The results were compared to age- and sex-adjusted reference values for healthy controls.

**Results:**

Both sexes reveal lower than zero *Z*-scores for lower leg 66% total cortical bone cross-sectional area to muscle cross-sectional area ratio (−0.97 ± 1.02, *p* = 0.002517 and −0.98 ± 1.40, *p* = 0.007050, respectively) while tibia 4% trabecular bone density *Z*-score was lowered in boys (−0.67 ± 1.20, *p* = 0.02259). In boys in Tanner stage 5 bone mass and dimensions were diminished in comparison to Tanner stages 3 and 4, while in girls, such a phenomenon was not observed. Similarly, bone formation and resorption were decreased in boys but not in girls. Consistently, bone turnover markers correlated positively with bone size, dimensions, and strength in boys only.

**Conclusions:**

T1DM patients revealed a decreased ratio of cortical bone area/muscle area, reflecting disturbed adaptation of the cortical shaft to the muscle force. When analyzing bone mass and dimensions, boys in Tanner stage 5 diverged from “less-mature” individuals, which may suggest that bone development in these individuals was impaired, affecting all three: mass, size, and strength. Noted in boys, suppressed bone metabolism may result in impairment of bone strength because of inadequate repair of microdamage and accumulation of microfractures.

## 1. Introduction

The type 1 diabetes mellitus is a chronic systemic autoimmune-mediated disease characterised by the insulin deficiency and hyperglycaemia [1, 2]. In most cases, the disease develops during childhood or early adolescence, and therefore, patients are exposed to the deleterious effects of the insulin and insulin-like growth factor deficiency for a long time [3–5]. The effect concerns not only bone mass, density, and fracture risk [6–9] but also may involve the linear growth of long bones [10]. Possible mechanisms involve hyperglycaemia, insulin deficiency, GH/IGF-1 axis disturbance, Wnt/*β*-catenin pathway alteration, decreased irisin secretion, and, probably, RANKL/RANK/OPG pathway perturbation [3–5, 11].

Our research group has observed significantly lower bone mineral density and mass (as measured by DXA) in adolescents with T1DM compared with age- and sex-adjusted healthy counterparts [12]. However, the dual-energy X-ray absorptiometry (DXA) measures bone mineral density as a areal bone mineral density (2D), thus cannot account for bone geometry and depth [13]. The peripheral quantitative computed tomography (pQCT) is able to provide a separate measurements of the cortical and trabecular bone as well as bone geometry and muscle cross-sectional area, utilizing low radiation dosage [14–17]. Since the bone measurement results interpretation may be considered as incomplete without taking into account the muscle mass [18] it is beneficial that all pQCT outcomes (including muscle) can be assessed by a single measurement.

Studies on lower leg in children with type 1 diabetes by pQCT have generated conflicting results [19–23], and most of the studies published so far focused only on a selected features of the bone. It seems advantageous to measure all currently available outcomes on all relevant slices, diverse in the meaning of the growth rate and the modelling of the bone. It would be relevant to incorporate into the analysis sex and Tanner stage, too, since they are ones of the key factors of the bone development.

An additional information about growth, modelling, and remodelling processes can be gathered by the bone turnover marker level measurement. Several bone turnover markers, which reflect the bone resorption and formation processes, have been described [24]. Osteocalcin (OC) is the most abundant bone noncollagenous protein produced by the osteoblast and thus reflects osteoblastic function and bone formation process [25]. The beta-isomer of the C-terminal telopeptide of type 1 collagen (CTx) is a fragment released from the telopeptide (end) region of type 1 collagen following its enzymatic degradation and can be detected in the circulation as a bone resorption marker [26]. Despite of its usefulness, it should be stressed that it is difficult to study bone metabolism in children/adolescents due to overlapping processes of the growth, modelling, and remodelling. Moreover, the most of the studies included the children/adolescents at different stages of puberty and, therefore, at different stages of acquisition of the bone mass. Along with the use of different bone formation and resorption markers as well as the assays, it may be the reason of the lack of concordant results about bone turnover marker level in diabetic children and adolescents [27]. Taking these into consideration, it seems to be reasonable to study diabetic bone with using both bone turnover markers and peripheral quantitative computed tomography.

The objectives of the study were to evaluate bone mineral density, mass, and geometry using peripheral quantitative computed tomography as well as bone turnover markers in the patients with type 1 diabetes mellitus.

## 2. Material and Methods

### 2.1. Studied Group

The group of 35 children (15 girls) aged from 12.34 to 17.95 yrs were recruited from the patients treated in the Department of Endocrinology and Diabetology. The inclusion criteria were as follows: age 12-18 yrs, diagnosis of diabetes mellitus type 1 according to International Society for Pediatric and Adolescent Diabetes criteria, duration of diabetes, and medical services received in the clinic for at least six months. All individuals were treated by the continuous subcutaneous insulin infusion. The exclusion criteria were as follows: the history of any acute (severe hypoglycaemia, diabetic ketoacidosis) or chronic (retinopathy, neuropathy, nephropathy, bone pain, or fracture) complications of diabetes, the presence of any associated metabolic bone or musculoskeletal diseases, and any chronic illness other than diabetes as well as any medications other than insulin. Finally, three individuals with Tanner stage 2 were excluded from the study due to their incompatibility to the entire group. The characteristics of the studied group are presented in Tables 1 and 2.

The study was conducted according to the Declaration of Helsinki and with a permission of the local Ethics Committee (Warsaw, Poland). Informed written consents were obtained from the parents or legal guardians of the participants.

### 2.2. Peripheral Quantitative Computed Tomography

Lower leg bone and muscle measurements were done with the Stratec XCT 2000L (Stratec Medizintechnik, Pforzheim, Germany) apparatus, software ver. 6.20, on nondominant leg [28]. Dominance was determined by the participant's report. The measurement sites were 4%, 14%, 38%, and 66% of the length of the tibia [28]. The tibia length was measured with the ruler from the middle of the inner ankle to the tibial plateau [28]. The scout view was used to determine the start position as follows: if the growth plate was visible, the reference line was placed in the middle of the growth plate; if the growth plate had fused, the reference line was placed in the middle of the distal end of the tibia. The scan lines were automatically placed at a distances of 4%, 14%, 38%, and 66% of the tibia length, proximal to the reference line. Scan speed, slice thickness, and voxel size were 20 mm/s, 2.3 mm, and 0.4 × 0.4 mm, respectively [28]. At the 4% site trabecular volumetric bone mineral density (mg/cm^3^), total volumetric bone mineral density (mg/cm^3^) and total bone cross-sectional area (mm^2^) were measured with using the CALCBD analysis algorithm, contour mode 1, peel mode 1, and threshold of 181 mg/cm^3^. Area was set as 45% (central) for trabecular volumetric bone mineral density determination [28]. At the 14% and 38% sites, the CORTBD algorithm with separation mode 1 and threshold of 711 mg/cm^3^ was used for determining cortical volumetric bone mineral density (mg/cm^3^) and cortical cross-sectional area (mm^2^), while threshold 280 mg/cm^3^ was used for polar strength strain index (mm^3^) calculation. The same threshold (280 mg/cm^3^) with the contour mode 1 and peel mode 1 was used for total bone cross-sectional area (mm^2^) determination [28]. At the 66% site, the CALCBD algorithm was used, with threshold of 40 mg/cm^3^, contour mode 3, peel mode 1, and filter F03F05 for muscle+bone area; threshold 280 mg/cm^3^ and contour mode1, and peel mode 2 for bone area [28]. Muscle cross-sectional area (mm^2^) was calculated by the subtraction of bone cross-sectional area from muscle+bone cross-sectional area. The bone mass per 1 running centimetre of bone in the particular slice was calculated from the density and cross-sectional area [28]. Outer cortical bone circumference, inner cortical bone circumference, and cortical shell thickness were calculated basing on the circular ring model [29]. Finally, the following ratios were calculated: tibia 14% cortical bone cross-sectional area to tibia 4% total bone cross-sectional area and tibia 4% bone mass to tibia 38% bone mass as a measures of the longwise bone shape [28] and lower leg 66% total cortical cross-sectional area to muscle cross-sectional area as a measure of the bone/muscle relationship [30, 31].

The effective doses involved in the procedure are as follows: scout view: 0.08 microSv; CT scans at 4%, 14%, 38, and 66% sites: 0.88 microSv (4 × 0.22 microSv); total dose: 0.96 microSv [28].

All measurements were done by the same operator on the same unit. The quality of each slice was rated from 1 (no movement) to 5 (extreme movement) by the same operator, according to the visual scale [32]. Slices rated >3 were excluded from the analysis as suggested by the others [32]. In the case of 4% and 14% of the tibia length, no exclusion was done; 1 exclusion were done for 38% site as well as for 66% site. The routine quality assurance procedures were carried out, basing on the phantom supplied by the manufacturer. The phantom comprises two “parts”: standard and cone. The standard phantom was measured each day when patients were measured. The cone phantom was measured monthly. Measurement errors were (CV%, standard phantom) 0.35% for total density, 0.44% for trabecular density, and 0.37% for cortical density in the study period.

### 2.3. Anthropometry

Body height (cm) and weight (kg) were measured in the standing position using stadiometer with medical scale (Tryb, Bydgoszcz, Poland). Body mass index (kg/m^2^) was calculated as body weight divided by squared height. Age of each participant was calculated from birth and examination dates.

### 2.4. Tanner Stage

The Tanner stage was assessed by physicians as a part of the routine diagnostic procedure.

### 2.5. Biochemistry

Blood samples were collected between 7:00 a.m. and 9:00 a.m. after an overnight fasting. HbA1c levels were analyzed using a direct turbidimetric inhibition immunoassay that determines HbA1c as a percentage of the total haemoglobin. The mean HbA1c level was defined as a mean value from the last year (for individuals with a diabetes duration of one year or longer) or a mean value from the 3 last measurements (for individuals with a diabetes duration shorter than one year). For evaluation of the bone formation and resorption, serum osteocalcin (OC) and carboxyterminal cross-linked telopeptide of type 1 collagen (CTx) concentrations were measured using ELECSYS N-MID Osteocalcin and ELECSYS beta-CrossLaps/serum-automated chemiluminescence assays (CLIA), respectively (Roche Diagnostics, Basel, Switzerland; CV ≤ 6.5% for OC and CV ≤ 4.7% for CTx, in our lab).

### 2.6. Statistics

*Z*-scores were calculated using LMS Growth v. 2.77 (Medical Research Council, UK), for height, weight, and body mass index basing on Polish reference data [33]; for pQCT basing on local reference data [34]; and for bone turnover markers basing on group of 158 healthy children and adolescents.

The Shapiro-Wilk test was used for assessing departures of analysed variables from Gaussian distribution. Normally distributed variables were presented as mean and standard deviation while nonnormally distributed as median and quartiles (Q1 and Q3). The one sample *t* test or Wilcoxon single signed rank test was used for the comparisons of *Z*-scores with the hypothetical mean zero. The two sample *t* test or Mann–Whitney test was used for the comparisons of two groups. In the case of *t* test, variances were compared with Levene test, and Welch correction was applied if needed. One-way ANOVA with Tukey post-test or Kruskal-Wallis nonparametric ANOVA was used for comparisons of *Z*-scores of pQCT outcomes by Tanner stage. Pearson coefficient of correlation was used to assess relationships between *Z*-scores of bone turnover markers and *Z*-scores of pQCT outcomes. Statistica v. 10 (StatSoft Inc., Tulsa, USA) was used for statistical calculations. *p* value less than 0.05 was considered as significant.

## 3. Results

The peripheral quantitative computed tomography outcomes were measured, and *Z*-scores were calculated according to age and sex for each participant. The mean *Z*-scores for all outcomes were compared with the hypothetical mean value of zero, separately for both sexes. The differences of *Z*-scores between girls and boys were tested simultaneously. The results were presented in Table 3. In the case of departure from Gaussian distribution, nonparametric tests were used, and median and quartiles were presented, too. In girls, *Z*-scores were significantly lower than 0 for tibia 14% cortical bone cross-sectional area and for ratio of lower leg 66% total cortical bone cross-sectional area to muscle cross-sectional area, with values of −0.65 ± 0.81 (*p* = 0.0079) and −0.97 ± 1.02 (*p* = 0.0025), respectively. In boys, lowered *Z*-scores were observed for tibia 4% trabecular bone density (−0.67 ± 1.20; *p* = 0.023) and for lower leg 66% total cortical bone cross-sectional area to muscle cross-sectional area ratio (−0.98 ± 1.40; *p* = 0.0070). On the contrary, for four outcomes in boys, mean *Z*-scores were heightened: 0.68 ± 0.84, *p* = 0.0019 for tibia 14% cortical bone density; 0.49 ± 1.00, *p* = 0.046 for tibia 38% cortical bone density; 0.66 ± 1.13, *p* = 0.018 for tibia 14% polar SSI; and 0.75 ± 1.21, *p* = 0.015 for lower leg 66% muscle cross-sectional area. Girls presented significantly higher *Z*-scores than boys for tibia 4% trabecular bone density 0.51 ± 1.15 vs. −0.67 ± 1.20, *p* = 0.0063 while for tibia 14% cortical bone density, tibia 14% cortical bone cross-sectional area and tibia 14% polar SSI girls show significantly lower *Z*-scores than boys: 0.08 ± 0.69 vs. 0.68 ± 0.84, *p* = 0.030; −0.65 ± 0.81 vs. −0.06 ± 0.82, *p* = 0.042; and −0.16 ± 1.13 vs. 0.66 ± 1.13, *p* = 0.042, respectively.

**Figure 1:**
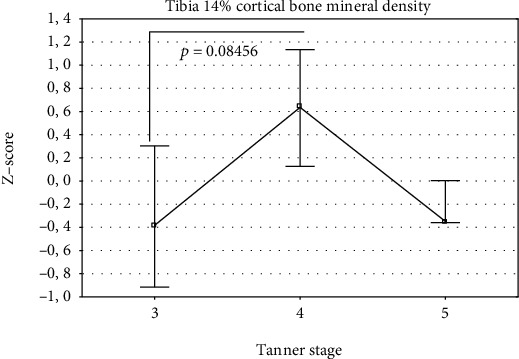
*Z*-scores for tibia 14% cortical bone mineral density in girls by Tanner stage (median and Q1-Q3). Overall Kruskal-Wallis ANOVA *p* is 0.0462; however, post-test does not reach significance level; the lowest *p* value is presented.

**Figure 2:**
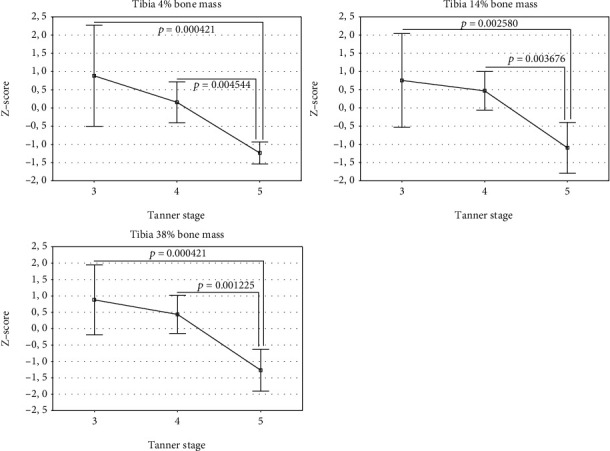
Lower leg *Z*-scores for bone masses in boys by Tanner stage (mean and 95% CI).

**Figure 3:**
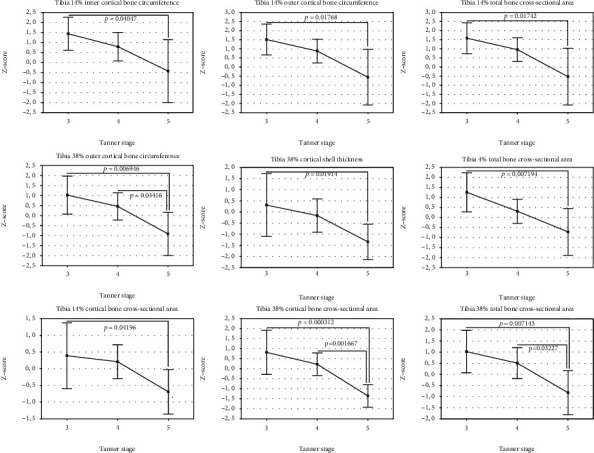
Lower leg *Z*-scores for selected cross-sectional bone dimensions in boys by Tanner stage (mean and 95% CI).

**Figure 4:**
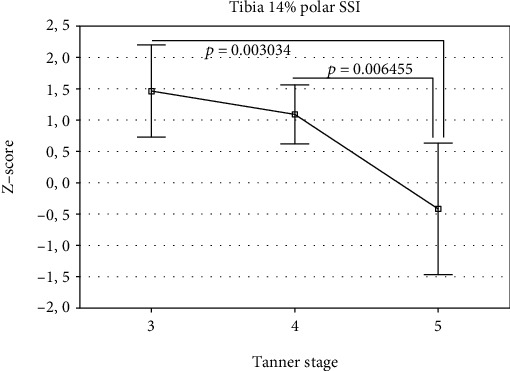
*Z*-scores for tibia 14% polar SSI in boys by Tanner stage (mean and 95% CI).

**Figure 5:**
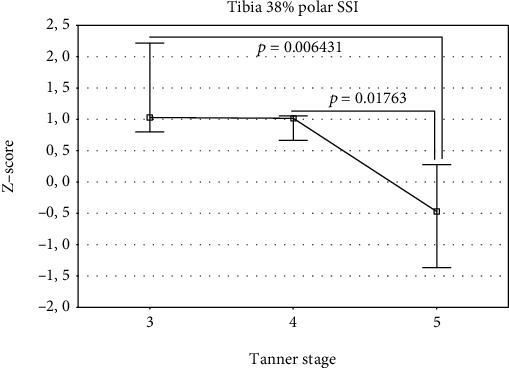
*Z*-scores for tibia 38% polar SSI in boys by Tanner stage (median and Q1-Q3).

The studied group was divided into 3 groups according to the Tanner stages 3, 4, and 5 (Table 2). Differences in the *Z*-scores between these groups were analyzed using one-way analysis of variance (ANOVA). The overall ANOVA results are shown in Tables 4 and 5 for girls and boys, respectively. If overall *p* value was less than 0.05, individual differences between the groups were assessed using the Bonferroni post-test. The results were presented in Figures 1–5. In the case of departure from Gaussian distribution Kruskal-Wallis nonparametric ANOVA with post-test (if applicable) was used. In girls, statistically significant differences between Tanner stage group were observed for tibia 14% cortical bone density, only. The overall *p* value (Kruskal-Wallis ANOVA) was 0.046. The biggest difference was noted between Tanner stages 4 and 5. Median (and Q1-Q3) *Z*-scores were as follows: 0.64 (0.13–1.14) and -0.35 (-0.36–0.00), respectively; however, post-test did not reach significance level. The lowest *p* value is 0.0846 for Tanner stage 3 versus Tanner stage 4 group. In boys, there were statistically significant differences for bone masses, cross-sectional bone dimensions, and strength strain indexes. For all three bone masses (4%, 14%, and 38% of the tibia length) Tanner stage 5 group had statistically significantly lower *Z*-scores than Tanner stage 3 and 4 group. Mean *Z*-scores for Tanner stage 5 were: −1.23 ± 0.33, −1.10 ± 0.75, and −1.27 ± 0.69, *p* value were (overall ANOVA) 0.00098, 0.0011, and 0.00016 for 4%, 14%, and 38% site, respectively. For bone cross-sectional dimensions, lower *Z*-scores were observed for Tanner stage 5, with exception of tibia 38% inner cortical bone circumference and tibia 14% cortical shell thickness, where no statistically significant differences were observed. For remainder of bone dimension outcomes, *Z*-scores were from −0.43 ± 1.70 for tibia 14% inner cortical bone circumference to −1.36 ± 0.61 for tibia 38% cortical bone cross-sectional area; *p* values (overall ANOVA) were from 0.040 to 0.00012, respectively. In the case of strength strain indexes (14% and 38% site), Tanner stage 5 boys had lower *Z*-score values than others, too. *Z*-score values were (mean ± SD) −0.42 ± 1.13 and (median Q1-Q3) -0.47 (-1.37–0.28), respectively, with *p* values (overall ANOVA) 0.0016 and 0.0028, despite of that in the case of the last one, nonparametric ANOVA was used.

Osteocalcin and C-terminal telopeptide levels were measured, and *Z*-scores were calculated for each individual (Table 6). In girls, *Z*-scores for both bone turnover markers did not differ significantly from zero; however, *Z*-scores for CTx tended to be higher than zero (*p* < 0.1) while *Z*-scores for OC were close to zero. In boys, *Z*-scores were lower than zero for both osteocalcin and C-terminal telopeptide. *Z*-score values were −0.64 ± 0.67 with *p* = 0.00040 and −0.68 ± 0.59 with *p* = 0.000099, respectively. Simultaneously, C-terminal telopeptide *Z*-scores in boys differed significantly from these in girls (−0.68 ± 0.59 vs. 0.66 ± 1.36, *p* = 0.0031) while *Z*-scores for osteocalcin did not.

The correlation analysis was carried out to establish relationships between *Z*-scores of bone turnover markers and *Z*-scores of pQCT outcomes. Coefficients of correlations (*r*) were calculated as well as *p* values and presented in Tables 7 and 8 for girls and boys, respectively. In girls, no significant correlations between *Z*-scores for osteocalcin and C-terminal telopeptide and pQCT outcomes were noted (*r* from -0.38 to 0.44; *p* > 0.05). In boys, osteocalcin *Z*-scores correlated significantly and negatively with *Z*-scores for 4% total bone density and 38% cortical bone density, *r* = −0.49, *p* = 0.029 and *r* = −0.53, *p* = 0.021, respectively. C-terminal telopeptide *Z*-scores correlated significantly and negatively with *Z*-scores for cortical bone density at 14% and 38% length of the tibia, with *r* equal -0.47 and -0.57 and *p* equal 0.044 and 0.014, respectively. Positive correlations were observed for *Z*-scores of inner cortical bone circumference at 14% and 38% site (*r* = 0.67, *p* = 0.0016 and *r* = 0.63, *p* = 0.0051, respectively), outer cortical bone circumference at the same sites (*r* = 0.72, *p* = 0.00054 and *r* = 0.70, *p* = 0.0013, respectively), total bone cross-sectional area at 4%, 14%, and 38% sites (*r* = 0.58, *p* = 0.0086, *r* = 0.72, *p* = 0.00057, and *r* = 0.69, *p* = 0.0014, respectively), strength strain indexes at 14% and 38% sites (*r* = 0.59, *p* = 0.0076 and *r* = 0.66, *p* = 0.0027, respectively), and for lower leg 66% total cortical bone cross-sectional area to muscle cross-sectional area ratio (*r* = 0.53, *p* = 0.023).

Correlation coefficients were computed to examine the correlations between *Z*-scores of bone turnover markers and mean HbA_1c_ level and presented in Table 9. In girls, osteocalcin level *Z*-score correlated negatively with mean HbA_1c_ level, while *Z*-score for CTx did not show significant correlation, as well as *Z*-scores for both bone turnover markers in boys.

## 4. Discussion

Until now, 5 studies concerning tibia bone measurement by pQCT in children with diabetes mellitus type 1 have been published [19–23]. All studies concerned tibial shaft, although different measurement sites were utilized: 38% [20], 50% [21], and/or 66% [19, 20, 22, 23] of the tibia length. Heap et al. [19], Moyer-Mileur et al. (2008) [20], and Saha et al. [21] reported the same cortical bone mineral density in patients with T1DM as in controls while Moyer-Mileur et al. (2004) [22] and Maratova et al. [23] showed higher cortical bone mineral density in patients than in controls. In our study, we observed higher cortical bone mineral density too; however, the finding concerns boys, only. In girls, cortical density remains unchanged. Bone mass was decreased in T1DM patients according to Moyer-Mileur et al. (2004) [22] and Saha et al. [21] while Heap et al. [19] and Moyer-Mileur et al. (2008) [20] did not note alterations. In our group, we did not observe alterations, too. Among the measures of bone geometry, only cortical bone area was studied by all authors. Moyer-Mileur et al. (2004) [22] and Saha et al. [21] noted decrease of cortical bone area while Heap et al. [19], Moyer-Mileur et al. (2008) [20], and Maratova et al. [23] did not note such decrease, the same as we. Moyer-Mileur et al. (2004) [22] also noted decrease of the cortical thickness as well as Maratova et al. [23] while Moyer-Mileur et al. (2008) [20], as well as we, did not note decrease. Total bone cross-sectional area remained unaltered in all papers studied this outcome [20, 23] as well as in our group. Marrow cavity size was studied by Moyer-Mileur et al. (2008) [20], and it remained unchanged. Since marrow cavity area may be treated as a surrogate of the inner cortical bone circumference, comparison with our cortical bone dimensions can be done. In our study, we did not observe alteration of cortical bone dimensions; both inner cortical bone circumference and outer cortical bone circumference were similar in DMT1 and healthy children. Bone strength was determined in 4 studies [20–23], whereby Moyer-Mileur et al. (2004) [22] and Maratova et al. [23] observed decrease of SSI polar while Moyer-Mileur et al. (2008) [20] and Saha et al. [21] found no difference, as well as we do, despite of the fact that Saha et al. [21] assessed polar section modulus instead of polar SSI. Bone mineral density at the 4% of the tibia length site was studied by 5 authors [19–23]. Saha et al. [21] and Moyer-Mileur et al. (2008) [20] did not note difference between T1DM patients and healthy ones while Heap et al. [19], Moyer-Mileur et al. (2004) [22], and Maratova et al. [23] observed decreased bone mineral density in T1DM, although the last one only in boys subgroup. Similarly, we observed decreased bone mineral density in boys, while in girls, a decrease was not observed. Such defect associated with low bone turnover presented only in trabecular bone was observed by Gunczler et al. [35] at the lumbar spine. Bone mass at this site was decreased according to Saha et al. [21] and Moyer-Mileur et al. (2004) [22], while according to Heap et al. [19], values were not altered. The same was observed in our group. Accordingly, 4% total bone area was lowered by Saha et al. [21] while our data present no alterations as well as for 4% total density, which was not studied by the others. Muscle area and cortical to muscle area ratios were described by Moyer-Mileur et al. (2004) [22] and Moyer-Mileur et al. (2008) [20]. According to Moyer-Mileur et al. (2008) [20], muscle area was not altered while Moyer-Mileur et al. (2004) [22] found muscle area in T1DM elevated. In our patients, muscle area was elevated in boys; in girls, upraising was slightly visible, however did not reach statistical significance level. Physical activity is a component of diabetes management, so such increasing is not unlikely. Simultaneously, cortical bone cross-sectional area to muscle cross-sectional was decreased according to Moyer-Mileur et al. (2004) [22] as well as in our data. 14% of the tibia length site was not studied up to date by the others, as well as longitudinal shape indexes. We observed no alterations for bone mass, inner and outer cortical bone circumference, cortical shell thickness, and total bone area in T1DM children as well as for tibia 4% bone mass to tibia 38% bone mass ratio and tibia 14% cortical bone cross-sectional area to tibia 4% total bone cross-sectional area ratio. On the contrary, we showed higher values for cortical bone density and SSI polar in T1DM boys (with no alteration in girls) while cortical bone cross-sectional area was decreased in T1DM girls; boys did not show such decrease.

Interestingly, our T1DM boys showed increased cortical bone mineral density for both sites: 14% and 38% of tibia length. Similar phenomenon was noted by Moyer-Mileur et al. (2004) [22] and Maratova et al. [23] for the 38% site. In our data, increase is even larger in the 14% site than in the 38% site; unfortunately, 14% site was not studied by the others. Simultaneously, we observed trend to diminishing cortical bone shell thickness, trend to increase inner cortical bone circumference, and total bone area. It may suggest impairment in metaphyseal inwaisting process with decreased endosteal apposition of bone and decreased periosteal resorption [36, 37]. It is consistent with negative correlations between cortical bone density and both bone turnover markers: OC and CTx, observed in male patient. Hygum et al. proposed that bone mineral density may be augmented in diabetic patients because of decreased bone turnover but not an intact mineralization process [38]. Secondary to low activity of metaphyseal inwaisting process, increase of total bone area and increase of bone mineral density seem to be the case of observed increase of SSI polar at the 14% of the tibia length site. Nonetheless, low bone turnover may result in impairment of bone strength because of inadequate repair of microdamage and accumulation of microfractures [39]. It is worth to be stressed that in the case of 38% site, in which bone is subject to modelling much longer, such pronounced alterations were not observed; polar SSI showed no alteration in our data as well in the case of others: Moyer-Mileur et al. (2008) [20] and Saha et al. [21]. It may suggest that observed increase of bone mineral density and SSI polar is a nonphysiological nature.

Concurrently, we observed increase of muscle cross-sectional area in children with T1DM and considerable decrease of total cortical bone cross-sectional area to muscle cross-sectional area ratio, totalled nearly -1 SD. It may suggests impairment of bone adaptation to loads from the muscle [30, 31, 40].

In the previously published studies, sexual maturation was primarily treated as cofactors [19, 20, 22]. From these, only Heap et al. [19] conducted separate analysis of impact of sexual maturation on bone. They showed that Tanner stage correlated positively with tibia 4% trabecular bone mineral density and tibia 66% cortical bone mineral density. In our group of patients, we did not see such dependency. Merely, in girls, trabecular bone mineral density showed trend to be lower along with Tanner stage, although without reaching statistical significance level. On the contrary, cortical bone mineral density at 14% of the tibia showed relationship with Tanner stage number. Surprisingly, the highest values were for Tanner stage 4, while for Tanner stages 3 and 5, values were lower. In boys with T1DM, bone masses, bone dimensions as well as polar SSI *Z*-scores reduced with increasing Tanner stage number. In the same phenomenon, we observed for forearm 66% bone mass, outer cortical bone circumference, cortical, and total bone cross-sectional areas as well as for SSI polar in the our group of patients [41]. It may suggest presence of incremental with maturation deficit of bone mass, size, and strength in boys with T1DM.

Our analyses revealed that *Z*-scores for OC and CTx were significantly lower in boys with T1DM, as compared to age-matched reference values, indicating a suppressed bone formation and resorption, respectively. Similar decreasing for both bone formation and resorption was observed previously by the others in children and adolescents (boys and girls analyzed together) [42–47]. However, the pooled analysis of systematic review revealed that only OC levels were significantly lower amongst T1DM children and adolescents, whereas the difference in CTx was insignificant [48]. On the other hand, a meta-analysis evaluating bone turnover markers in both T1DM and T2DM, children and adults, also reported decreased OC and CTx in diabetes [49]. Similarly, Hygum et al. concluded in systematic review and meta-analysis that both bone resorption and formation are lower in diabetic patients regardless of age and diabetes type as indicating by consistently lower levels of CTx and OC in diabetes compared with controls [38].

A possible mechanism of low bone turnover is hyperglycaemia [38]. Hyperglycaemia affects the skeleton at both cellular and extracellular bone matrix levels [50]. In vitro, hyperglycaemia decreases osteoclast and osteoblast function and may thus lead to decreased bone turnover [51, 52]. At the tissue level, hyperglycaemia affects the organic bone matrix through the accumulation of advanced glycation end products (AGEs) incorporated into bone by nonenzymatic glycation of collagen leading to inferior bone strength and disrupting the adhesion of osteoblasts to the extracellular matrix [53, 54]. Pooled correlation analysis of systematic review showed a significant negative correlation between OC and metabolic control in children and adolescents with T1DM, indicating that an increase in HbA_1c_ reduces bone formation [48]. Furthermore, a human study concluded that OC is associated with improved glucose tolerance and insulin secretion [55]. In several animal studies, OC have demonstrated positive effects on both insulin production, insulin release, and insulin sensitivity [56, 57]. With this knowledge, it would be expected to find a lower HbA_1c_ in individuals with high OC *Z*-scores. In our study, we noticed a significant negative correlation between OC *Z*-score and HbA_1c_ in girls.

According to our knowledge, only two studies [7, 22] has examined bone metabolism using bone turnover markers and bone status by pQCT. Bechtold et al. [7] did not find any correlation between bone turnover markers and pQCT outcomes. However, they studied upper extremity and different bone turnover markers than we do. Lower extremity was studied by Moyer-Mileur et al. (2004) [22]; unfortunately, correlation analysis between bone turnover markers and pQCT outcomes was not carried out. Few studies investigated the bone geometry using another techniques as magnetic resonance imaging [45], radiography [58], digitalized X-rays [59], and bone mass by DXA [43]. Pater et al. noticed that bone mass measured by DXA correlated well and positively with OC [43]. CTx was found to inversely associate with bone mass by magnetic resonance imaging [45]. Multiple regression analysis of Franceschi et al. showed that inner diameter measured by digitalized X-rays at the level of the 2nd metacarpal bone was influenced positively only by bone formation marker P1NP (not by BAP and CTx) [59], whereas we noted the positive correlation between bone resorption (CTx) and inner cortical bone circumference in boys. We did not note any correlation between bone formation marker and bone geometry parameters, probably because OC is rather marker of bone mineralization than bone matrix production [42].

Summarising of the results of our work by sex, it seems that only boys present impairment in metaphyseal inwaisting process with increased cortical density; reduced bone masses, bone dimensions, and polar SSI with increasing Tanner stage number. Additionally, observed in boys but not in girls, correlations between the bone turnover markers and the pQCT bone parameters may suggest that decreased level of bone metabolism may be connected with increased cortical bone density and that high level of bone resorption markers (indicator of bone modelling) may be attributed to increased bone size and strength. Taking into consideration that other papers [7, 60, 61] do not always show difference between sexes and that in our group, levels of bone turnover markers vary substantially between the sexes, we hypothesise that at least in our group of patients, the levels of bone turnover markers may be more important factor than sex to maintain proper bone density, size, and strength. However, it is possible that this phenomenon may be related to relatively small number of patients which is the main limitation of the presented study.

## 5. Conclusions

Type 1 diabetes mellitus patients revealed a decreased ratio of cortical bone area/muscle area, reflecting disturbed adaptation of the cortical shaft to the muscle force. When analyzing bone mass and dimensions, boys in Tanner stage 5 diverged from “less mature” individuals, which may suggest that bone development in these individuals was impaired, affecting all three: mass, size, and strength. Noted in boys, suppressed bone metabolism may result in impairment of bone strength because of inadequate repair of microdamage and accumulation of microfractures.

## Figures and Tables

**Table 1:** Characteristics of the studied group by sex.

Gaussian distributed variables					
	Female (*n* = 15)		Male (*n* = 20)		*p* value^1)^
	Mean	SD	Mean	SD	
Height (cm)	158.11	6.59	177.05	8.23	2.134∗10^−8^
Weight (kg)	53.35	9.95	67.12	7.59	5.167∗10^−5^
BMI (kg/m^2^)	21.26	3.30	21.40	1.90	0.8859^∗^
*Z*-score height	-0.62	0.97	0.46	0.99	**0.002902**
*Z*-score weight	0.12	0.78	0.37	0.53	0.2425
*Z*-score BMI	0.44	0.79	0.24	0.63	0.3978
HbA_1c_ mean (%)	7.61	0.85	7.55	1.45	0.8858^∗^
Osteocalcin (microg/ml)	61.60	31.64	67.05	27.28	0.5885
C-terminal telopeptide (ng/ml)	1.012	0.485	1.015	0.327	0.9856

Nonnormally distributed variable					
	Female (*n* = 15)		Male (*n* = 20)		*p* value^2)^
	Median	Quartiles (Q1-Q3)	Median	Quartiles (Q1-Q3)	
Age (yrs)	14.6	12.9-17.2	16.4	14.5-17.6	0.2433
Age at diagnosis (yrs)	10.7	8.8-13.0	12.0	8.5-14.6	0.4432
Time since diagnosis (yrs)	4.9	1.8-6.2	2.9	1.2-7.2	0.7003

BMI: body mass index; HbA1c: glycated haemoglobin. ^1)^Student's *t* test. ∗Student's *t* test with Welch correction for nonequal variances. ^2)^Mann–Whitney test.

**Table 2:** Number of individuals by Tanner stage and sex.

Sex	Tanner stage 3	Tanner stage 4	Tanner stage 5	Sum
Female	6	6	3	15
Male	5	8	7	20
Sum	11	14	10	Overall *n* = 35

**Table 3:** Peripheral quantitative computed tomography *Z*-scores in comparison with hypothetical mean zero by sex.

	Female (*n* = 15) Mean (SD) *p* value (diff. from 0)	Male (*n* = 20) Mean (SD) *p* value (diff. from 0)^1)^	*p* value (diff. between female and male)
Bone mineral densities:			
*Z*-score tibia 4% trabecular bone density	0.51 (1.15) (*p* = 0.1088)	-0.67 (1.20) **(p** = 0.02259**)**	**0.006321**
*Z*-score tibia 4% total bone density	0.11 (1.33) (*p* = 0.2375)^∗^	-0.40 (1.23) (*p* = 0.1594)	0.1134^∗∗^
*Z*-score tibia 14% cortical bone density	0.08 (0.69) (*p* = 0.6763)	0.68 (0.84) **(p** = 0.001883**)**	**0.03013**
*Z*-score tibia 38% cortical bone density	0.03 (1.16) (*p* = 0.2271)^∗^	0.49 (1.00) **(p** = 0.04570**)**	0.5099^∗∗^

Bone masses:			
*Z*-score tibia 4% bone mass	-0.07 (0.84) (*p* = 0.7642)	-0.15 (1.10) (*p* = 0.5537)	0.8108
*Z*-score tibia 14% bone mass	-0.37 (0.88) (*p* = 0.1226)	-0.01 (1.11) (*p* = 0.9758)	0.3032
*Z*-score tibia 38% bone mass	-0.33 (1.26) (*p* = 0.3226)	-0.07 (1.17) (*p* = 0.7834)	0.5394

Cross-sectional dimensions:			
*Z*-score tibia 14% inner cortical bone circumference	0.00 (1.32) (*p* = 0.9959)	0.52 (1.36) (*p* = 0.1010)	0.2635
*Z*-score tibia 38% inner cortical bone circumference	0.39 (0.93) (*p* = 0.1255)	0.60 (1.27) (*p* = 0.0538)	0.5941
*Z*-score tibia 14% outer cortical bone circumference	-0.06 (1.30) (*p* = 0.8510)	0.54 (1.39) (*p* = 0.1007)	0.2032
*Z*-score tibia 38% outer cortical bone circumference	-0.05 (1.07) (*p* = 0.8720)	0.11 (1.20) (*p* = 0.7035)	0.7036
*Z*-score tibia 14% cortical shell thickness	-0.48 (1.16) (*p* = 0.1342)	-0.47 (1.14) (*p* = 0.0784)	0.9920
*Z*-score tibia 38% cortical shell thickness	-0.77 (1.45) (*p* = 0.0582)	-0.47 (1.12) (*p* = 0.0822)	0.5010
*Z*-score tibia 14% cortical bone cross-sectional area	-0.65 (0.81) **(p** = 0.007881**)**	-0.06 (0.82) (*p* = 0.7464)	**0.04167**
*Z*-score tibia 38% cortical bone cross-sectional area	-0.44 (1.36) (*p* = 0.2289)	-0.20 (1.14) (*p* = 0.4443)	0.5860
*Z*-score tibia 4% total bone cross-sectional area	-0.08 (1.10) (*p* = 0.7755)	0.19 (1.20) (*p* = 0.4913)	0.4986
*Z*-score tibia 14% total bone cross-sectional area	-0.05 (1.31) (*p* = 0.8861)	0.59 (1.41) (*p* = 0.0753)	0.1779
*Z*-score tibia 38% total bone cross-sectional area	-0.03 (1.07) (*p* = 0.9045)	0.15 (1.15) (*p* = 0.5699)	0.6316

Longitudinal shape indexes:			
*Z*-score tibia 4% bone mass/tibia 38% bone mass	0.39 (0.94) (*p* = 0.1336)	-0.08 (0.79) (*p* = 0.6605)	0.1248
*Z*-score tibia 14% cortical bone cross-sectional area/tibia 4% total bone cross-sectional area	-0.31 (1.08) (*p* = 0.2890)	-0.23 (1.05) (*p* = 0.3474)	0.8228

Strength strain index:			
*Z*-score tibia 14% polar SSI	-0.16 (1.13) (*p* = 0.5881)	0.66 (1.13) **(p** = 0.01761**)**	**0.04163**
*Z*-score tibia 38% polar SSI	-0.19 (1.26) (*p* = 0.5752)	0.35 (1.28) (*p* = 0.2461)	0.2286

Muscle and bone:			
*Z*-score lower leg 66% muscle cross-sectional area	0.67 (1.23) (*p* = 0.0550)	0.75 (1.21) **(p** = 0.01478**)**	0.8429
*Z*-score lower leg 66% total cortical bone cross-sectional area/muscle cross-sectional area	-0.97 (1.02) **(p** = 0.002517**)**	-0.98 (1.40) **(p** = 0.007050**)**	0.9802

^∗^Wilcoxon single signed rank test; medians and Q1–Q3 are 0.24 (-0.46–1.32) for *Z*-score tibia 4% total bone density and 0.44 (-0.41–0.94) for *Z*-score tibia 38% cortical bone density. ^∗∗^Mann-Whitney test; medians and Q1–Q3 are 0.44 (-0.41–0.94) in female and 0.40 (-0.21–1.27) in male for *Z*-score tibia 4% total bone density and *Z*-score tibia 38% cortical bone density, respectively, ^1)^for 38% and 66% sites *n* = 19 as well as for *Z*-score tibia 4% bone mass to tibia 38% bone mass ratio.

**Table 4:** *Z*-scores of pQCT outcomes by Tanner stage in girls.

	Tanner stage 3 Mean (SD) (*n* = 6)	Tanner stage 4 Mean (SD) (*n* = 6)	Tanner stage 5 Mean (SD) (*n* = 3)	ANOVA overall *p* value
Bone mineral densities				
*Z*-score tibia 4% trabecular bone density	0.14 (1.14)	0.80 (1.37)	0.68 (0.82)	0.6200
*Z*-score tibia 4% total bone density	-0.72 (1.63)	0.77 (0.70)	0.43 (0.96)	0.1326
*Z*-score tibia 14% cortical bone density	-0.33 (0.64)	0.63 (0.51)	-0.23 (0.21)	**0.04620** ^∗^
*Z*-score tibia 38% cortical bone density	-0.26 (1.18)	0.32 (1.40)	0.05 (0.79)	0.4665^∗^

Bone masses				
*Z*-score tibia 4% bone mass	-0.17 (1.00)	-0.01 (0.84)	0.01 (0.76)	0.9713^∗^
*Z*-score tibia 14% bone mass	-0.79 (0.88)	-0.09 (0.85)	-0.09 (0.86)	0.3358
*Z*-score tibia 38% bone mass	-0.67 (1.42)	0.03 (1.45)	-0.39 (0.32)	0.6959^∗^

Cross-sectional dimensions				
*Z*-score tibia 14% inner cortical bone circumference	0.44 (1.58)	-0.28 (1.42)	-0.32 (0.12)	0.5633
*Z*-score tibia 38% inner cortical bone circumference	0.54 (1.11)	0.54 (0.75)	-0.21 (0.96)	0.4912
*Z*-score tibia 14% outer cortical bone circumference	0.17 (1.50)	-0.21 (1.53)	-0.25 (0.35)	0.8642
*Z*-score tibia 38% outer cortical bone circumference	-0.16 (1.10)	0.24 (1.28)	-0.37 (0.67)	0.7106^∗^
*Z*-score tibia 14% cortical shell thickness	-1.15 (1.45)	-0.07 (0.84)	0.06 (0.44)	0.2562
*Z*-score tibia 38% cortical shell thickness	-1.08 (2.02)	-0.59 (1.22)	-0.52 (0.55)	0.8186
*Z*-score tibia 14% cortical bone cross-sectional area	-1.22 (0.85)	-0.34 (0.50)	-0.13 (0.73)	0.0699
*Z*-score tibia 38% cortical bone cross-sectional area	-0.76 (1.69)	-0.13 (1.42)	-0.42 (0.33)	0.7556
*Z*-score tibia 4% total bone cross-sectional area	0.45 (1.27)	-0.51 (0.95)	-0.30 (0.89)	0.3244
*Z*-score tibia 14% total bone cross-sectional area	0.20 (1.50)	-0.19 (1.53)	-0.27 (0.33)	0.8461
*Z*-score tibia 38% total bone cross-sectional area	-0.14 (1.10)	0.25 (1.28)	-0.38 (0.67)	0.7106^∗^

Longitudinal shape indexes				
*Z*-score tibia 4% bone mass/tibia 38% bone mass	0.67 (1.09)	0.09 (0.84)	0.41 (0.97)	0.6050
*Z*-score tibia 14% cortical bone cross-sectional area/tibia 4% total bone cross-sectional area	-1.05 (1.27)	0.25 (0.66)	0.06 (0.49)	0.0800

Strength strain indexes				
*Z*-score tibia 14% polar SSI	-0.54 (0.98)	0.04 (1.46)	0.18 (0.67)	0.5984
*Z*-score tibia 38% polar SSI	-0.29 (1.02)	0.07 (1.69)	-0.50 (0.98)	0.6015^∗^

Muscle and bone				
*Z*-score lower leg 66% muscle cross-sectional area	0.35 (1.72)	1.10 (0.89)	0.44 (0.57)	0.5725
*Z*-score lower leg 66% total cortical bone cross-sectional area/muscle cross-sectional area	-0.91 (1.12)	-1.01 (1.27)	-0.99 (0.38)	0.8119^∗^

^∗^Kruskal-Wallis nonparametric ANOVA. Medians (Q1–Q3) are *Z*-score tibia 14% cortical bone density: -0.38 (-0.92–0.31); 0.64 (0.13–1.14); and -0.35 (-0.36–0.00). *Z*-score tibia 38% cortical bone density: 0.02 (-0.7–0.5); 0.71 (0.38–1.19); and -0.40 (-0.41–0.96). *Z*-score tibia 4% bone mass: -0.43 (-0.57–-0.19); -0.17 (-0.71–0.86); and 0.26 (-0.84–0.61). *Z*-score tibia 38% bone mass: -0.7 (-1.18–0.16); 0.08 (-1.08–0.72); and -0.57 (-0.58–-0.02). *Z*-score tibia 38% inner cortical bone circumference: 0.31 (-0.34–1.83); 0.35 (0.02–1.21); and 0.31 (-1.32–0.38). *Z*-score tibia 38% total bone cross-sectional area: -0.48 (-0.66–-0.35); 0.19 (-0.75–1.04); and -0.25 (-1.11–0.21). *Z*-score tibia 38% polar SSI: -0.51 (-0.59–-0.39); 0.11 (-1.38–0.99); and -0.60 (-1.42–0.54). *Z*-score lower leg 66% total cortical bone cross-sectional area/muscle cross-sectional area: -1.22 (-1.41–-0.92); -0.84 (-1.24–-0.23); and -0.83 (-1.42–-0.71) for Tanner stages 3, 4, and 5, respectively.

**Table 5:** *Z*-scores of pQCT outcomes by Tanner stage in boys.

	Tanner stage 3 Mean (SD) (*n* = 5)	Tanner stage 4 Mean (SD) (*n* = 8)^1)^	Tanner stage 5 Mean (SD) (*n* = 7)	ANOVA overall *p* value
Bone mineral densities:				
*Z*-score tibia 4% trabecular bone density	0.08 (0.88)	-0.44 (0.71)	-1.46 (1.49)	0.0640
*Z*-score tibia 4% total bone density	-0.54 (0.77)	-0.16 (1.07)	-0.58 (1.71)	0.7897
*Z*-score tibia 14% cortical bone density	0.23 (0.95)	0.57 (0.71)	1.12 (0.80)	0.1790
*Z*-score tibia 38% cortical bone density	0.43 (0.98)	0.43 (0.71)	0.60 (1.35)	0.9437

Bone masses:				
*Z*-score tibia 4% bone mass	0.88 (1.12)	0.16 (0.67)	-1.23 (0.33)	**0.0009802**
*Z*-score tibia 14% bone mass	0.75 (1.04)	0.47 (0.63)	-1.10 (0.75)	**0.001080**
*Z*-score tibia 38% bone mass	0.88 (0.86)	0.44 (0.63)	-1.27 (0.69)	**0.0001557**

Cross-sectional dimensions:				
*Z*-score tibia 14% inner cortical bone circumference	1.44 (0.67)	0.79 (0.85)	-0.43 (1.70)	**0.04027**
*Z*-score tibia 38% inner cortical bone circumference	1.06 (1.18)	0.63 (0.98)	0.25 (1.63)	0.58621
*Z*-score tibia 14% outer cortical bone circumference	1.52 (0.68)	0.88 (0.78)	-0.56 (1.65)	**0.01573**
*Z*-score tibia 38% outer cortical bone circumference	1.03 (0.76)	0.47 (0.74)	-0.91 (1.16)	**0.005885**
*Z*-score tibia 14% cortical shell thickness	-0.47 (1.13)	-0.43 (1.19)	-0.53 (1.27)	0.98808
*Z*-score tibia 38% cortical shell thickness	0.31 (1.14)	-0.16 (0.81)	-1.34 (0.86)	**0.01675**
*Z*-score tibia 14% cortical bone cross-sectional area	0.39 (0.79)	0.21 (0.61)	-0.69 (0.72)	**0.02635**
*Z*-score tibia 38% cortical bone cross-sectional area	0.82 (0.89)	0.22 (0.61)	-1.36 (0.61)	**0.0001242**
*Z*-score tibia 4% total bone cross-sectional area	1.26 (0.79)	0.31 (0.72)	-0.72 (1.26)	**0.009007**
*Z*-score tibia 14% total bone cross-sectional area	1.58 (0.68)	0.96 (0.78)	-0.53 (1.68)	**0.01513**
*Z*-score tibia 38% total bone cross-sectional area	1.02 (0.77)	0.51 (0.75)	-0.82 (1.07)	**0.005877**

Longitudinal shape indexes:				
*Z*-score tibia 4% bone mass/tibia 38% bone mass	0.15 (1.12)	-0.06 (0.52)	-0.27 (0.82)	0.6767
*Z*-score tibia 14% cortical bone/tibia 4% total bone cross-sectional area	-0.88 (0.67)	-0.14 (0.92)	0.15 (1.29)	0.2450

Strength strain indexes:				
*Z*-score tibia 14% polar SSI	1.46 (0.59)	1.09 (0.57)	-0.42 (1.13)	**0.001606**
*Z*-score tibia 38% polar SSI	1.39 (0.84)	0.79 (0.53)	-0.83 (1.20)	**0.00280** ^∗^

Muscle and bone:				
*Z*-score lower leg 66% muscle cross-sectional area	1.59 (0.80)	0.82 (1.35)	0.08 (1.01)	0.0957
*Z*-score lower leg 66% total cortical bone cross-sectional area/muscle cross-sectional area	-0.39 (1.08)	-0.71 (1.65)	-1.67 (1.21)	0.2515

^1)^For 38% and 66% sites *n* = 7 as well as for *Z*-score tibia 4% bone mass to tibia 38% bone mass ratio. ^∗^Kruskal-Wallis nonparametric ANOVA. Medians (Q1–Q3) are 1.03 (0.8–2.21); 1.01 (0.66–1.06); -0.47 (-1.37–0.28) for Tanner stages 3, 4, and 5, respectively.

**Table 6:** *Z*-scores for bone turnover markers in comparison with hypothetical mean zero by sex.

	Female: mean (SD) *p* value (diff. from 0)	Male: mean (SD) *p* value (diff. from 0)	*p* value (diff. between female and male)
Osteocalcin (micro g/ml)	-0.17 (0.91) 0.4799 (*n* = 15)	-0.64 (0.67) **(p** = 0.0003994**)**(*n* = 20)	0.0865
C-terminal telopeptide (ng/ml)	0.66 (1.36) 0.0903 (*n* = 14)	-0.68 (0.59) **(p** = 0.00009919**)**(*n* = 19)	**0.003083** ^∗^

^∗^Student's *t* test with Welch correction for nonequal variances.

**Table 7:** Correlations of *Z*-scores of bone turnover markers and *Z*-scores of pQCT outcomes in girls.

	*Z*-score OC		*Z*-score CTx	
	*r*	*p* value	*r*	*p* value
Bone mineral densities				
*Z*-score tibia 4% trabecular bone density	0.16	0.5604	-0.13	0.6533
*Z*-score tibia 4% total bone density	-0.01	0.9674	-0.24	0.4137
*Z*-score tibia 14% cortical bone density	-0.08	0.7657	-0.38	0.1795
*Z*-score tibia 38% cortical bone density	-0.24	0.3953	-0.21	0.4764

Bone masses				
*Z*-score tibia 4% bone mass	0.00	0.9977	0.03	0.9275
*Z*-score tibia 14% bone mass	-0.21	0.4419	-0.20	0.4876
*Z*-score tibia 38% bone mass	-0.22	0.4248	-0.26	0.3774

Cross-sectional dimensions				
*Z*-score tibia 14% inner cortical bone circumference	-0.16	0.5632	-0.08	0.7852
*Z*-score tibia 38% inner cortical bone circumference	-0.35	0.2025	-0.16	0.5750
*Z*-score tibia 14% outer cortical bone circumference	-0.25	0.3741	-0.06	0.8283
*Z*-score tibia 38% outer cortical bone circumference	-0.31	0.2539	-0.28	0.3364
*Z*-score tibia 14% cortical shell thickness	0.11	0.6888	0.03	0.9232
*Z*-score tibia 38% cortical shell thickness	0.01	0.9782	-0.11	0.7104
*Z*-score tibia 14% cortical bone cross-sectional area	-0.09	0.7595	-0.07	0.8181
*Z*-score tibia 38% cortical bone cross-sectional area	-0.15	0.5843	-0.23	0.4194
*Z*-score tibia 4% total bone cross-sectional area	0.02	0.9345	0.23	0.4283
*Z*-score tibia 14% total bone cross-sectional area	-0.25	0.3745	-0.06	0.8399
*Z*-score tibia 38% total bone cross-sectional area	-0.31	0.2547	-0.27	0.3440

Longitudinal shape indexes				
*Z*-score tibia 4% bone mass/tibia 38% bone mass	0.28	0.3115	0.44	0.1170
*Z*-score tibia 14% cortical bone cross-sectional area/tibia 4% total bone cross-sectional area	-0.06	0.8335	-0.21	0.4757

Strength strain indexes				
*Z*-score tibia 14% polar SSI	-0.32	0.2491	-0.28	0.3323
*Z*-score tibia 38% polar SSI	-0.28	0.3160	0.04	0.8886

Muscle and bone				
*Z*-score lower leg 66% muscle cross-sectional area	-0.16	0.5730	-0.26	0.3665
*Z*-score lower leg 66% total cortical bone cross-sectional area/muscle cross-sectional area	0.03	0.9289	0.08	0.7767

*r*: coefficient of correlation; OC: osteocalcin; CTx: C-terminal telopeptide.

**Table 8:** Correlations of *Z*-scores of bone turnover markers and *Z*-scores of pQCT outcomes in boys.

	*Z*-score OC		*Z*-score CTx	
	*r*	*p* value	*r*	*p* value
Bone mineral densities				
*Z*-score tibia 4% trabecular bone density	-0.42	0.0671	-0.27	0.2619
*Z*-score tibia 4% total bone density	-0.49	**0.02869**	-0.37	0.1137
*Z*-score tibia 14% cortical bone density	-0.23	0.3229	-0.47	**0.04409**
*Z*-score tibia 38% cortical bone density	-0.53	**0.02085**	-0.57	**0.01349**

Bone masses				
*Z*-score tibia 4% bone mass	0.04	0.8754	0.31	0.1997
*Z*-score tibia 14% bone mass	0.02	0.9222	0.34	0.1572
*Z*-score tibia 38% bone mass	-0.02	0.9241	0.36	0.1375

Cross-sectional dimensions				
*Z*-score tibia 14% inner cortical bone circumference	0.31	0.1771	0.67	**0.001606**
*Z*-score tibia 38% inner cortical bone circumference	0.32	0.1820	0.63	**0.005117**
*Z*-score tibia 14% outer cortical bone circumference	0.31	0.1900	0.72	**0.0005422**
*Z*-score tibia 38% outer cortical bone circumference	0.22	0.3609	0.70	**0.001296**
*Z*-score tibia 14% cortical shell thickness	-0.19	0.4270	-0.36	0.1288
*Z*-score tibia 38% cortical shell thickness	-0.12	0.6329	0.04	0.8771
*Z*-score tibia 14% cortical bone cross-sectional area	0.06	0.8098	0.25	0.3077
*Z*-score tibia 38% cortical bone cross-sectional area	0.00	0.9982	0.38	0.1244
*Z*-score tibia 4% total bone cross-sectional area	0.42	0.0677	0.58	**0.008588**
*Z*-score tibia 14% total bone cross-sectional area	0.31	0.1906	0.72	**0.0005687**
*Z*-score tibia 38% total bone cross-sectional area	0.22	0.3657	0.69	**0.001411**

Longitudinal shape index				
*Z*-score tibia 4% bone mass/tibia 38% bone mass	0.02	0.9197	-0.07	0.7735
*Z*-score tibia 14% cortical bone cross-sectional area/tibia 4% total bone cross-sectional area	-0.37	0.1087	-0.41	0.0827

Strength strain indexes				
*Z*-score tibia 14% polar SSI	0.19	0.4131	0.59	**0.007599**
*Z*-score tibia 38% polar SSI	0.18	0.4702	0.66	**0.002664**

Muscle and bone				
*Z*-score lower leg 66% muscle cross-sectional area	0.06	0.8158	0.01	0.9713
*Z*-score lower leg 66% total cortical bone cross-sectional area/muscle cross-sectional area	0.06	0.8064	0.53	**0.02337**

*r*: coefficient of correlation; OC: osteocalcin; CTx: C-terminal telopeptide.

**Table 9:** Correlations of *Z*-scores of bone turnover markers and mean HbA1c in girls and boys.

HbA_1c_ level (%)				
	Female		Male	
	*r*	*p* value	*r*	*p* value
*Z*-score OC	-0.56	**0.02870**	-0.02	0.9223
*Z*-score CTX	-0.35	0.2259	-0.19	0.4457

HbA_1c_: glycated haemoglobin; *r*: coefficient of correlation; OC: osteocalcin; CTx: C-terminal telopeptide.

## Data Availability

The numerical data used to support the findings of this study are available from the corresponding author upon reasonable request.
